# Some Secrets of Fluorescent Proteins: Distinct Bleaching in Various Mounting Fluids and Photoactivation of Cyan Fluorescent Proteins at YFP-Excitation

**DOI:** 10.1371/journal.pone.0018586

**Published:** 2011-04-07

**Authors:** Naila Malkani, Johannes A. Schmid

**Affiliations:** Department of Vascular Biology and Thrombosis Research, Center for Physiology and Pharmacology, Medical University Vienna, Vienna, Austria; Griffith University, Australia

## Abstract

**Background:**

The use of spectrally distinct variants of green fluorescent protein (GFP) such as cyan or yellow mutants (CFP and YFP, respectively) is very common in all different fields of life sciences, e.g. for marking specific proteins or cells or to determine protein interactions. In the latter case, the quantum physical phenomenon of fluorescence resonance energy transfer (FRET) is exploited by specific microscopy techniques to visualize proximity of proteins.

**Methodology/Principal Findings:**

When we applied a commonly used FRET microscopy technique - the increase in donor (CFP)-fluorescence after bleaching of acceptor fluorophores (YFP), we obtained good signals in live cells, but very weak signals for the same samples after fixation and mounting in commercial microscopy mounting fluids. This observation could be traced back to much faster bleaching of CFP in these mounting media. Strikingly, the opposite effect of the mounting fluid was observed for YFP and also for other proteins such as Cerulean, TFP or Venus. The changes in photostability of CFP and YFP were not caused by the fixation but directly dependent on the mounting fluid. Furthermore we made the interesting observation that the CFP-fluorescence intensity increases by about 10 - 15% after illumination at the YFP-excitation wavelength – a phenomenon, which was also observed for Cerulean. This photoactivation of cyan fluorescent proteins at the YFP-excitation can cause false-positive signals in the FRET-microscopy technique that is based on bleaching of a yellow FRET acceptor.

**Conclusions/Significance:**

Our results show that photostability of fluorescent proteins differs significantly for various media and that CFP bleaches significantly faster in commercial mounting fluids, while the opposite is observed for YFP and some other proteins. Moreover, we show that the FRET microscopy technique that is based on bleaching of the YFP is prone to artifacts due to photoactivation of cyan fluorescent proteins under these conditions.

## Introduction

Cyan and yellow fluorescent proteins (CFP and YFP, respectively) are very frequently used in a great variety of experiments employing fluorescence microscopy, because they can be distinguished quite easily by appropriate filter sets. Especially the original mammalian optimized versions, also known as enhanced cyan and yellow fluorescent proteins (ECFP and EYFP) are commonly applied, as they have been among the first spectrally distinct variants of green fluorescent protein [1]. Moreover, they are well suited for fluorescence resonance energy transfer (FRET) microscopy to visualize protein interactions or conformational changes [2], [3]. In that case, the energy of the donor fluorophore (CFP) is transferred to an acceptor fluorophore (YFP) by a dipole interaction resulting typically in a decrease of donor and an increase in acceptor fluorescence. This phenomenon can be visualized by various microscopy techniques. One of them monitors the increase of donor fluorescence after photobleaching of the acceptor, which eliminates the energy transfer [4]. This technique was originally developed for small molecular weight fluorophores such as Cy3 and Cy5 [5], but is also often applied to various fluorescent proteins [6], [7], [8], [9], [10]. It is practically done by acquiring a donor (e.g. CFP) image, followed by bleaching of the acceptor (e.g. YFP) with its specific excitation wavelength and the acquisition of a second donor image [11]. An increase in donor fluorescence, which is usually visualized by calculating a ratio or a difference image of the two donor images, indicates close proximity of FRET donor and acceptor proteins.

## Results and Discussion

**Figure 1:**
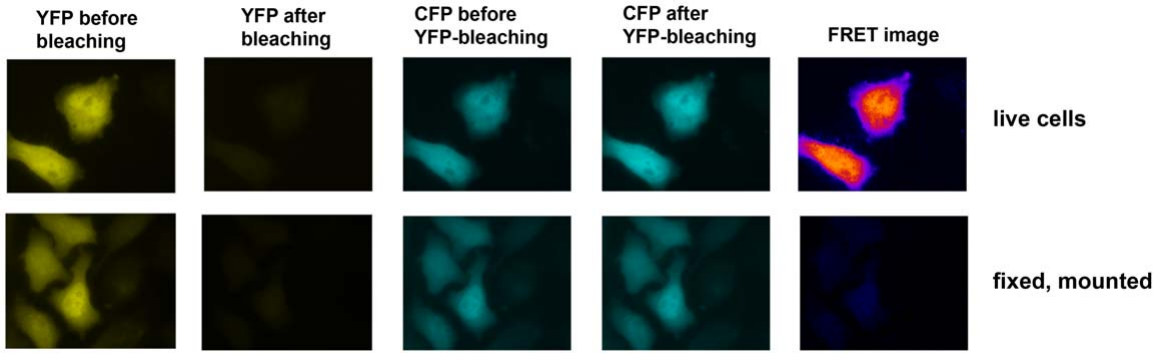
Donor recovery FRET microscopy of live and fixed cells. 293 cells were transfected with a CFP-YFP fusion protein (ECYFP) representing a positive FRET sample and imaged as described [31], either as live cells or after fixation and mounting with Dako Fluorescence Mounting Fluid. In brief, a CFP-image was acquired, followed by bleaching of YFP and acquisition of a second CFP-image. An increase in CFP-signal upon YFP-bleaching is indicated by a pseudo-colored difference image (FRET image), which appeared much more intense for live cells than for fixed, mounted cells imaged under the same conditions. The pseudocolored difference image is shown in a “fire” look-up table, which emphasizes the signal intensity differences between live and fixed cells. It does not show absolute values.

**Figure 2:**
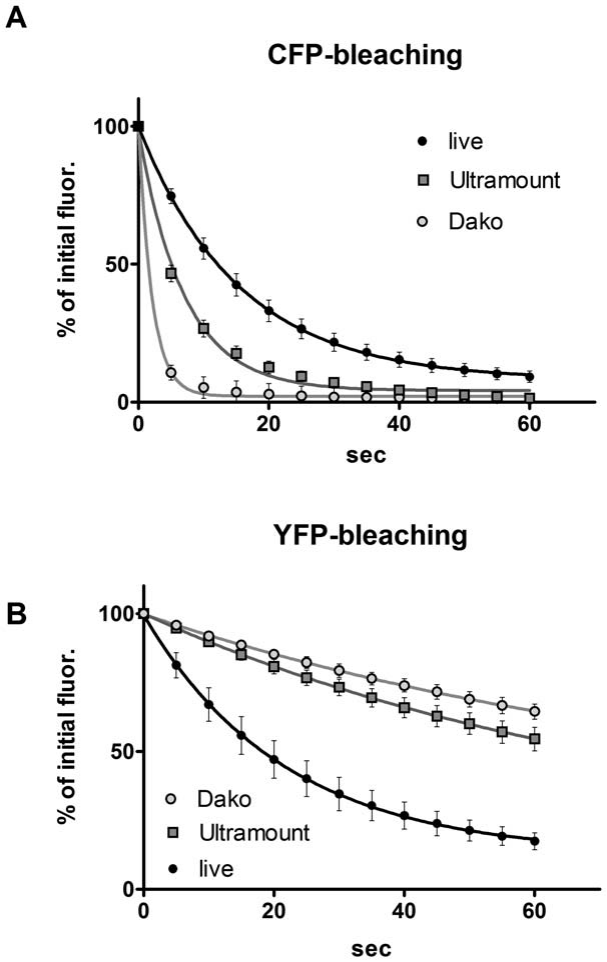
Differential bleaching kinetics of CFP and YFP in different fluids. 293 cells were transfected with either CFP (A) or YFP (B) and the bleaching of the fluorescent proteins was recorded either for live cells in medium or for fixed cells after mounting in Fisher Scientific Ultramount (Ultramount) or Dako Fluorescence Mounting Fluid (Dako). Bleaching was recorded on a Zeiss Axiovert 135 microscope using continuous illumination at the respective wavelength with 50% energy of a 100W Mercury-lamp. Metamorph™ 7.5 software was used to acquire images at 5 sec intervals. Camera: PhotometricsCoolsnap; exposure time: 50 msec. Data points are mean of n = 4. Error bars represent standard error of mean (SEM).

**Figure 3:**
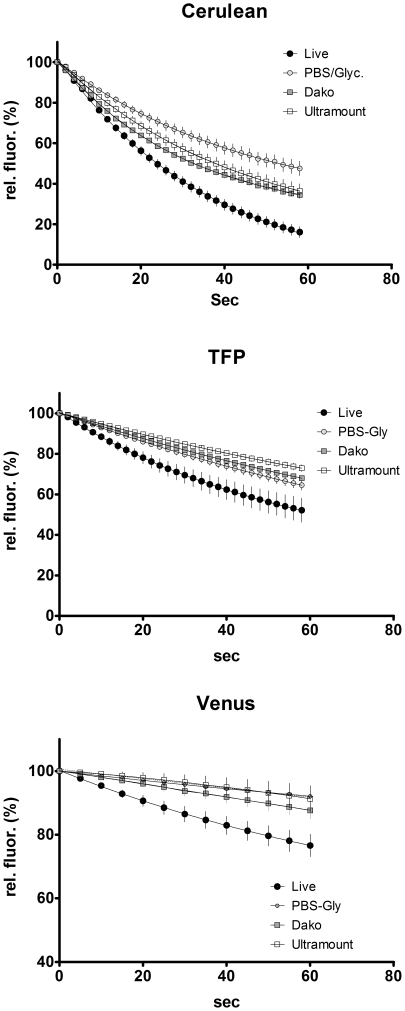
Bleaching kinetics of Cerulean, teal fluorescent protein (TFP) and Venus in various mounting fluids. 293 cells were transfected with the different fluorescent protein constructs and imaged either live or after fixation and mounting as specified in Fig. 2 with 2 sec time intervals. Data points are mean of n = 5; error bars represent SEM.

**Figure 4:**
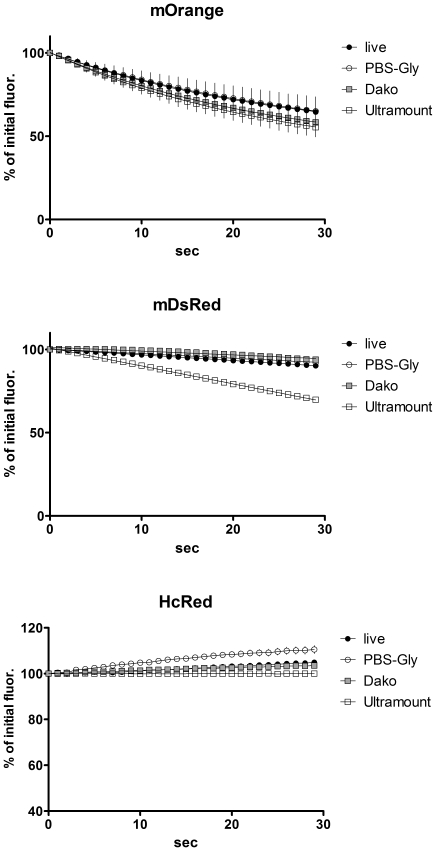
Bleaching kinetics of mOrange, mDsRed and HcRed1 in different mounting fluids. 293 cells were transfected with the different fluorescent protein constructs and imaged either live or after fixation and mounting as specified in Fig. 2. Time intervals: 1 sec. Data points are mean of n = 7; error bars represent SEM.

We applied the FRET microscopy technique that is based on bleaching of the acceptor to cells transfected with interacting CFP- and YFP-tagged proteins or a positive CFP-YFP FRET probe. Interestingly, we noticed a striking difference in results obtained with living cells and those generated with fixed cells after mounting them on coverslips with a commercial mounting fluid (Fig. 1). While the donor recovery was perfectly visible in living cells, we did not observe a significant increase in fixed cells. Moreover, we noticed that the donor fluorescence faded much faster in the mounted, fixed samples, while it appeared more difficult to bleach the acceptor. This prompted us to determine the kinetics of photobleaching of CFP and YFP in live versus fixed cells and to investigate the influence of the fluid, in which the cells are embedded for microscopy. After mounting the cells in a commercial mounting fluid (Dako Fluorescence Mounting Medium or Thermo Scientific Ultramount), the bleaching of CFP was much faster than in live cells at the same illumination intensity (Fig. 2A), whereas the bleaching of YFP was slowed down (Fig. 2B). Interestingly, the kinetics of bleaching hardly differed between live cells imaged in medium and cells that had been fixed with 4% paraformaldehyde and imaged in PBS (Fig. S1). This was observed for both CFP and YFP. Mounting of fixed cells in PBS/glycerol (1∶7) resulted in enhanced photostability of CFP, while bleaching kinetics of YFP was similar to live cells or fixed cells imaged in PBS alone (Fig. S1). The drastic differential effect of the mounting fluid on the bleaching kinetics of CFP and YFP stimulated us to investigate this aspect also for other fluorescent proteins. First, we focused our experiments on alternative cyan fluorescent proteins, such as Cerulean (an improved cyan variant, [12]) and mTFP1 (monomeric teal fluorescent protein 1, [13]) – as well as an improved version of yellow fluorescent protein, termed Venus [14]. Interestingly, in all of these cases mounting of cells expressing the different fluorescent proteins resulted in an increased photostability and reduced bleaching kinetics (Fig. 3) – similar to what had been observed for YFP – but contrary to the behavior of CFP. Next, we extended our experiments to a range of orange and red fluorescent proteins: mOrange [15]; mDsRed1, a monomeric red fluorescent protein from Clontech Inc. (Mountain View, CA, USA) and HcRed1 [16]. While we did not notice any significant effect of the mounting fluid for mOrange, we observed a destabilizing effect of the Ultramount fluid for mDsRed. Strikingly, HcRed did not show any bleaching but rather a slight increase in fluorescence upon prolonged illumination at its excitation wavelength, which was most pronounced when it was embedded in PBS/glycerol (Fig. 4). In none of these experiments, we observed any significant effect of the incubation time with the mounting fluid on the bleaching characteristics arguing against a penetration issue as cause for the observed differences. This is in line with the notion that glycerol, a frequently used component of mounting fluids, exhibits efficient permeation through lipid membranes, which occurs predominantly via aquaporins [17]. This seems to apply also to other common constituents of mounting fluids.

Next, we wanted to test whether the particular cellular environment influences the observed differences in bleaching kinetics. To that end, we repeated the experiments in a different cell type, which resulted in a very similar bleaching behavior (Fig. S2). This indicates that the observed differences in photostability are really due to distinct behavior of the various fluorescent proteins.

**Figure 5:**
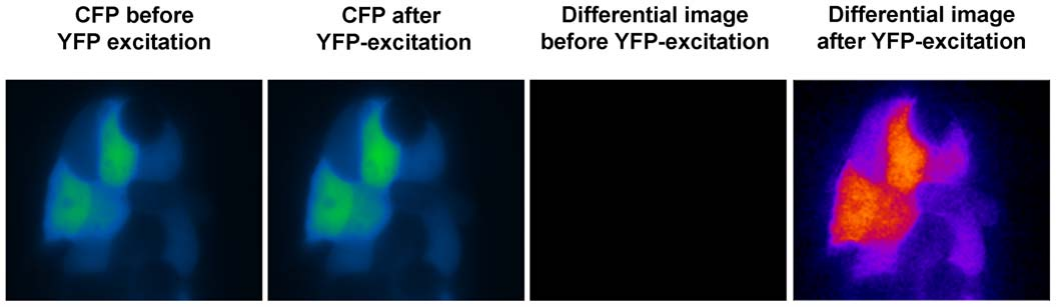
Photoactivation of CFP by illumination at the YFP-excitation wavelength on a conventional epifluorescence microscope. 293 cells were transfected with CFP and imaged as live cells on a conventional epifluorescence microscope (Zeiss Axiovert 135) with a standard CFP filter as specified in the Methods section. After the first CFP-image, the cells were excited with the YFP-specific excitation light (500 nm) for 5 sec, followed by another CFP-image with the same acquisition settings as the first one. The two images are presented in a “Green Fire Blue” pseudocolour lookup table using ImageJ software to emphasize the difference in fluorescence intensity (designated as CFP before and after YFP-excitation, respectively). A differential image was calculated with ImageJ for two CFP-images before YFP-excitation and for a CFP-image after YFP-excitation minus a CFP-image before YFP-excitation (Differential image after YFP-excitation). These two images are shown in a “Fire” lookup table to emphasize the difference between the two differential images.

**Figure 6:**
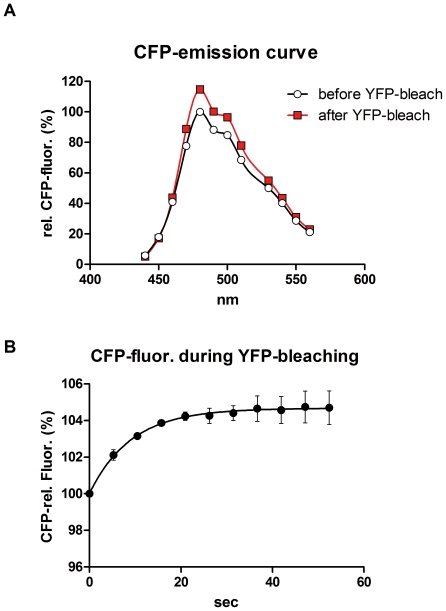
Photoactivation of CFP by illumination at the YFP-excitation wavelength on a confocal laser scanning microscope. A) 293 cells expressing CFP were fixed and mounted in PBS/glycerol followed by spectral imaging on a Leica TCS SP5 confocal microscope. Emission curves were acquired with 405 nm laser excitation before and after strong illumination of a region of interest with the 514 nm laser line (50 iterative scans with 50% laser intensity) to mimic bleaching of YFP. The fluorescence intensity was quantified for the region of interest and normalized to the CFP-peak before YFP-bleaching. B) Time course of CFP-fluorescence upon YFP-bleaching by laser scanning. 293 cells expressing CFP were fixed and mounted in PBS/glycerol (1∶7) followed by imaging on a Leica TCS SP5 laser scanning microscope. Cells were imaged repetitively with 405 nm excitation and CFP-filter sets interrupted by intermediate illumination of a region of interest with 514 nm laser (50% laser power, 50 iterative scans). The intensity of the CFP-fluorescence was quantified in this region and is shown as percentage of the initial fluorescence. Error bars represent SEM.

**Figure 7:**
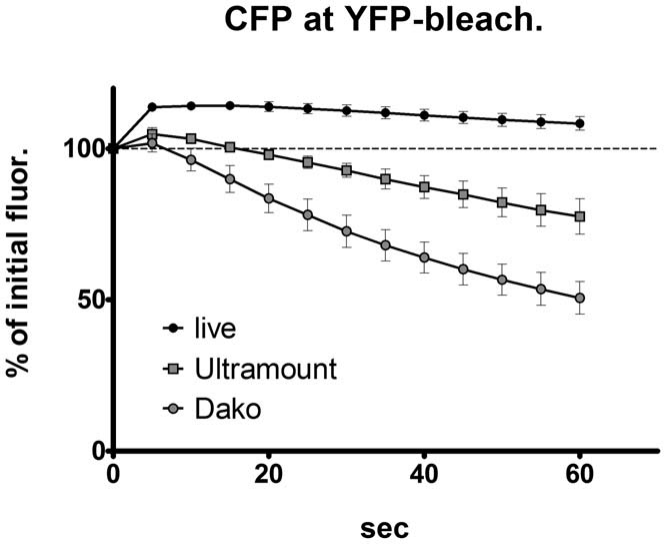
Time course of CFP-fluorescence upon YFP-bleaching by conventional microscopy in different fluids. 293 cells were transfected with CFP alone and the time course of CFP-fluorescence was recorded with intermediate repetitive YFP-excitation either for live cells in medium or for fixed cells after mounting in Fisher Scientific Ultramount (Ultramount) or Dako Fluorescence Mounting Fluid (Dako) using a conventional epifluorescence microscope (as described in Fig. 2). A computer-controlled Ludl filter wheel was applied to switch from the continuous YFP-excitation briefly to CFP-excitation for capturing a CFP-signal (camera: PhotometricsCoolsnap; exposure time: 50 msec). Data points are mean of n = 3; error bars represent SEM.

**Figure 8:**
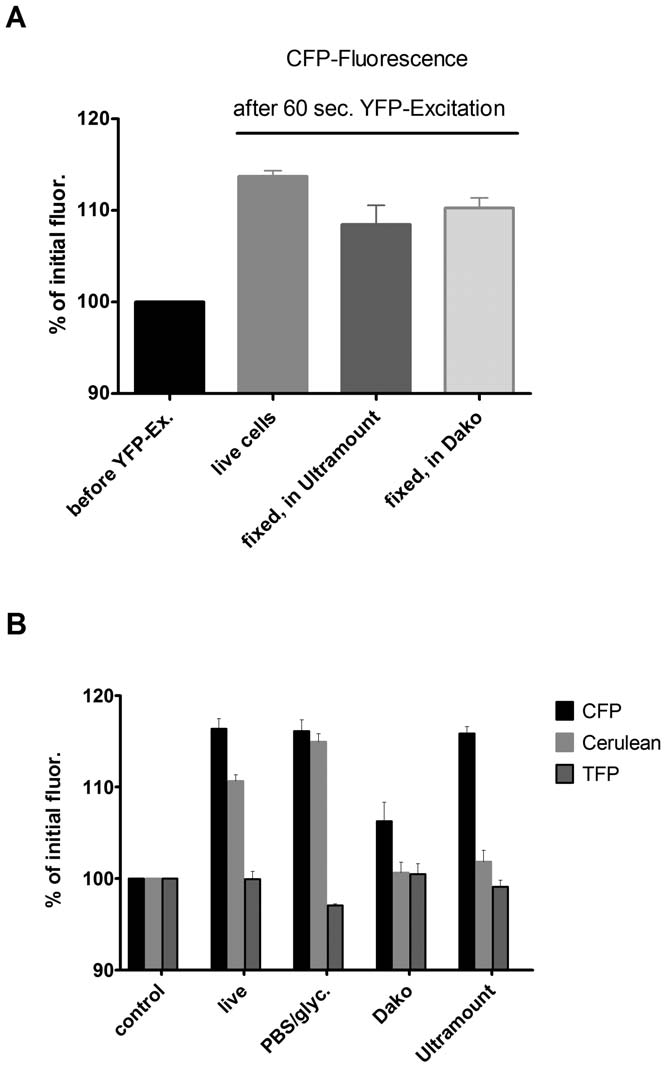
Increase of CFP- or Cerulean fluorescence by YFP-excitation in different fluids (without repetitive CFP-excitation). A) 293 cells expressing CFP alone were quickly imaged for CFP-fluorescence on a Zeiss Axiovert 135 microscope equipped with Ludl-filter wheels controlled by Metamorph 7.5-software, followed by an immediate switch to the YFP-excitation wavelength (500 nm) for 60 sec (50% power of a 100W mercury lamp) and the acquisition of a second CFP-image using the same image acquisition parameters. CFP-fluorescence is expressed as percentage of the initial fluorescence (before YFP-Ex.) and shown after YFP-excitation for live cells, fixed cells mounted in Ultramount or fixed cells mounted in Dako Fluorescence Mounting Fluid. B) 293 cells expressing either CFP, Cerulean or TFP alone were imaged quickly for cyan fluorescence as in A) followed by a fast switch to the YFP excitation wavelength for 5 sec (at 50% power of a 100W mercury lamp) and the acquisition of a second cyan fluorescence image. Data is shown as in A) for live cells or fixed cells mounted in PBS/glycerol (PBS/glyc.), Dako or Ultramount fluorescent mounting fluid as indicated. Error bars represent SEM; n = 7.

In addition to the drastic effect of the mounting fluid on the bleaching kinetics of fluorescent proteins, we made another striking observation: Illumination of CFP-expressing cells with the excitation wavelength of YFP, as it is done for the donor recovery method of FRET microscopy, resulted in a clear increase of the CFP-fluorescence signal even in the absence of any YFP (Fig. 5). This increase was furthermore detected by spectral imaging microscopy as an elevation of the emission peak without a significant change of the shape of the spectrum (Fig. 6A). The extent of donor fluorescence intensification was in a range of 5–15% of the initial fluorescence, which is a value that is quite common for donor recovery FRET microscopy and which would be equivalent to a FRET efficiency of 5–13% (based on the equation: efficiency E = 1 – F_DA_/F_D,_ where F_DA_ is donor fluorescence in presence of acceptor (before acceptor bleaching) and F_D_ is donor fluorescence in absence of a functional acceptor (after acceptor bleaching, [3]).When we recorded the time course of this increase of the CFP-signal during illumination at the YFP-excitation for fixed cells mounted in PBS/glycerol using confocal laser scanning microscopy, we observed a single exponential characteristic with a plateau (Fig. 6B). In live cells imaged by conventional epifluorescence microscopy a similar increase in CFP-fluorescence was observed, which was even faster occurring already within 5 sec of YFP-illumination. However, in fixed cells embedded in commercial mounting fluids, the CFP-fluorescence increased slightly for a brief period, followed by a considerable decrease (Fig. 7). This decrease could be attributed to the repeated short illumination at the CFP-excitation wavelength that was necessary to acquire the time course of CFP-fluorescence and was not caused by the illumination at the YFP-excitation wavelength. When we acquired a CFP-image followed by an immediate change to the YFP-excitation lasting for 60 sec and acquisition of a second CFP-image without any illumination at the CFP-excitation wavelength in between, we did not observe any reduction in CFP-fluorescence but again a rise of the CFP-signal (Fig. 8A). Nevertheless, the extremely fast bleaching of CFP in cells in commercial mounting fluids makes it quite difficult to preserve the CFP-fluorescence during manipulation of the microscope system – and therefore to observe a potential increase of the CFP-signal in FRET samples after bleaching of YFP in these embedded samples. This explains why the donor recovery FRET microscopy technique often does not work in these types of samples. Contrary to that this method can lead to false-positive FRET signals in live cells or in cells embedded in PBS/glycerol based on the fact that we observed an increase in CFP-fluorescence after illumination at the YFP-excitation wavelength, even when there was no YFP expressed. Strikingly, this phenomenon was also observed for the improved cyan fluorescent protein Cerulean; but not for teal fluorescent protein, TFP – and again the effect was influenced by the nature of the mounting fluid (Fig. 8B). We suppose that the photoactivation of cyan fluorescent proteins at YFP-excitation adds to the problem of photoconverting YFP into a CFP-like fluorescent protein upon bleaching of YFP which was first reported in 2005 [18], [19]. While this photoconversion could not be reproduced in some laboratories [20], [21], later studies confirmed that this phenomenon can occur [22] and that it depends on thermal activation, diffusivity, hydration and oxygenation [23] finally resulting in a significant variability of this phenomenon.

Yet, it can be concluded that FRET microscopy based on bleaching of YFP in a CFP/YFP-sample can lead to false-positive signals due to photoconversion of YFP into a CFP-like fluorophore and due to an increase of CFP-fluorescence under these conditions. Contrary to that, false-negative results are likely in samples embedded in commercial mounting fluids due to the fast bleaching of CFP in these media. As a consequence, this common technique of FRET microscopy should generally not be used for the CFP/YFP fluorophore pair but instead either 3-filter methods of FRET microscopy [2], [3] should be exploited or alternatives techniques such as fluorescence lifetime imaging (FLIM), which can be used to assess a FRET effect in a robust manner via the decrease of the donor fluorescence lifetime [24], [25], [26]. Another alternative is recording the kinetics of photobleaching of the donor, which is slowed done in presence of a FRET acceptor. This method, which was originally developed for low molecular weight fluorophores [27], was also shown to work with CFP/YFP protein pairs [28]. Overall, we can conclude that the complex photochemistry of fluorescent proteins has to be considered for sophisticated techniques such as FRET microscopy and that the drastic differential effect of the mounting fluid on bleaching kinetics of fluorescent proteins in general, should be taken into account also in other microscopy techniques.

## Materials and Methods

### Materials

Commercial mounting fluids were from Dako (Dako Fluorescent Mounting Fluid) or Thermo Scientific (Ultramount). CFP- and YFP-constructs (pECFP-C1 and p-EYFP-N1), as well as the monomeric DsRed (mDsRed) were from Clontech Inc (California, USA).

For generating the CFP-YFP fusion construct, we first cloned a yellow fluorescent protein coding sequence into a pEGFP-C1 vector (Clontech Inc.). This was done by PCR-amplification of the YFP (variant GFP10C, which was a generous gift of Dr. Roger Tsien, described in [29]) from a a pRSETB vector (Invitrogen, Carlsbad, CA, USA), using the pRSETB-primer (forward): 5′-AAA AAG ATC TGC TAG CGT CGA CAC CAT GGG TCG GGA TCT GTA C containing a Bgl II site and the reverse primer 5′-AAA AAA GCT TGC GGC CGC ATT TGT ATA GTT CAT CCA TGC C containing a Hind III site. The PCR product was cut with Bgl II and Hind III and cloned into a pEGFP-C1 vector (Clontech Inc.) that had been cut with the same restriction sites, resulting in a pEGFP-YFP fusion construct. Subsequently, the EGFP part was cut out with Age I and Bgl II and replaced by ECFP (obtained from pECFP-C1, Clontech Inc, cut with the same restriction sites). This resulted in a CFP-YFP fusion construct with a 27-amino acid spacer in between the CFP and the YFP-tag.

The alternative cyan fluorescent protein Cerulean was obtained from the Addgene plasmid repository (www.addgene.org), and the monomeric teal fluorescent protein, mTFP1, was obtained from Allele Biotechnology Inc (www.allelebiotech.com). The monomeric Orange fluorescent protein construct (mOrange) was from Roger Tsien's group and the HcRed1 construct from Karel Drbal. 293 cells (HEK-293) and the prostate cancer cell line DU145 were from the ATCC cell culture collection.

### Cell culture, transfections and mounting of cells

HEK-293 cells were cultured as described [30] and DU145 cells were cultured in Eagle's Minimum Essential Medium medium containing 2 mM glutamine, 10% FCS, 100 u/ml penicillin and 100 mg/ml streptomycin. One day before transfection, cells were seeded on round glass coverslips (15 mm diameter). HEK-293 cells were transfected with Lipofectamine, (Invitrogen, Carlsbad, CA, USA) or Fugene (Roche, Vienna, Austria) according to the manufacturers' protocols or with the calcium-phosphate precipitation method. DU145 cells were transfected with Exgen (Fermentas, St-Leon-Rot, Germany) as described by the manufacturer. One day after transfection, cells were examined either live or after fixation for 10 min in 4% paraformaldehyde in PBS and mounting in different fluids: 1) PBS, 2) PBS + glycerol (1∶7); 3) Dako Fluorescent mounting fluid; 4) Ultramount from Thermoscientific. Microscopy of live cells in medium or of fixed cells in PBS alone was done in a self-made sealed incubation chamber containing 90 µl of the respective solution. Microscopy of fixed and mounted cells was performed after mounting of the coverlips with the respective fluid on glass slides.

### Fluorescence microscopy

Conventional epifluorescence microscopy was either done with a Nikon Diaphot TMD microscope as described in [28] or with a Zeiss Axiovert 135 microscope equipped with a Photometrics Coolsnap camera. Fluorescence filter sets were from Chroma Technology Inc. (VT, USA). For cyan fluorescent proteins (CFP; Cerulean and mTFP1) the following set was used: Excitation: 436 nm (20 nm bandwidth), dichroic mirror: 455 nm longpass; emission: 480 nm (40 nm bandwidth). For yellow fluorescent proteins (YFP and Venus) this set was used: Excitation 500 nm (bandwidth 20 nm), dichroic mirror: 515 nm longpass, emission: 535 nm (bandwidth 30 nm). For mOrange, mDsRed and HcRed the following set was used: Excitation: 535 nm (50 nm bandwidth); dichroic mirror: 565 nm longpass; emission filter: 610 nm (70 nm bandwidth). Dichroic mirrors were mounted in the filter turret of the microscope; excitation and emission filters were mounted on Ludl filter wheels and controlled by Metamorph 7.5 software. Fluorescence Resonance Energy Transfer (FRET) microscopy was performed by the donor recovery after acceptor bleaching technique as described in [31]. Laser scanning microscopy and spectral imaging was performed on a Leica TCS SP5 confocal microscope as described in the figure legends.

### Statistics

Data points are mean of at least 4–5 samples. Error bars represent standard error of mean (SEM). Non-linear curve fitting was performed with GraphPad Prism 5.0™ using a single exponential decay equation.

### Ethics Statement

This study was conducted according to the principles expressed in the Declaration of Helsinki.

## Supporting Information

Figure S1 **Bleaching kinetics of CFP and YFP is not significantly altered by the fixation.** 293 cells were transfected with CFP (A) or YFP (B) and bleaching was recorded by conventional epifluorescence microscopy in live cells in medium; in 4% paraformaldehyde-fixed cells in PBS; in fixed cells mounted with Dako Fluorescent Mounting Fluid and in fixed cells mounted in PBS/glycerol (PBS/Glyc., 1∶7) as indicated.
(TIF)

Figure S2 **Bleaching characteristics is very similar in different cellular environments.** 293 or DU145 cells were transfected with CFP (upper panel) or teal fluorescent protein (TFP, lower panel) as indicated and bleaching was recorded for live cells or for cells fixed with 4% paraformaldehyde in PBS and mounted in PBS/glycerol 1∶7 (PBS-Gly); in Dako fluorescent mounting fluid or in Thermofisher Ultramount.
(TIF)
