# Supplementary figures and images for: Some Secrets of Fluorescent Proteins: Distinct Bleaching in Various Mounting Fluids and Photoactivation of Cyan Fluorescent Proteins at YFP-Excitation
Source: PLoS One. 2011 Apr 7;6(4):e18586. doi: 10.1371/journal.pone.0018586 (PMC3072413; doi:10.1371/journal.pone.0018586)

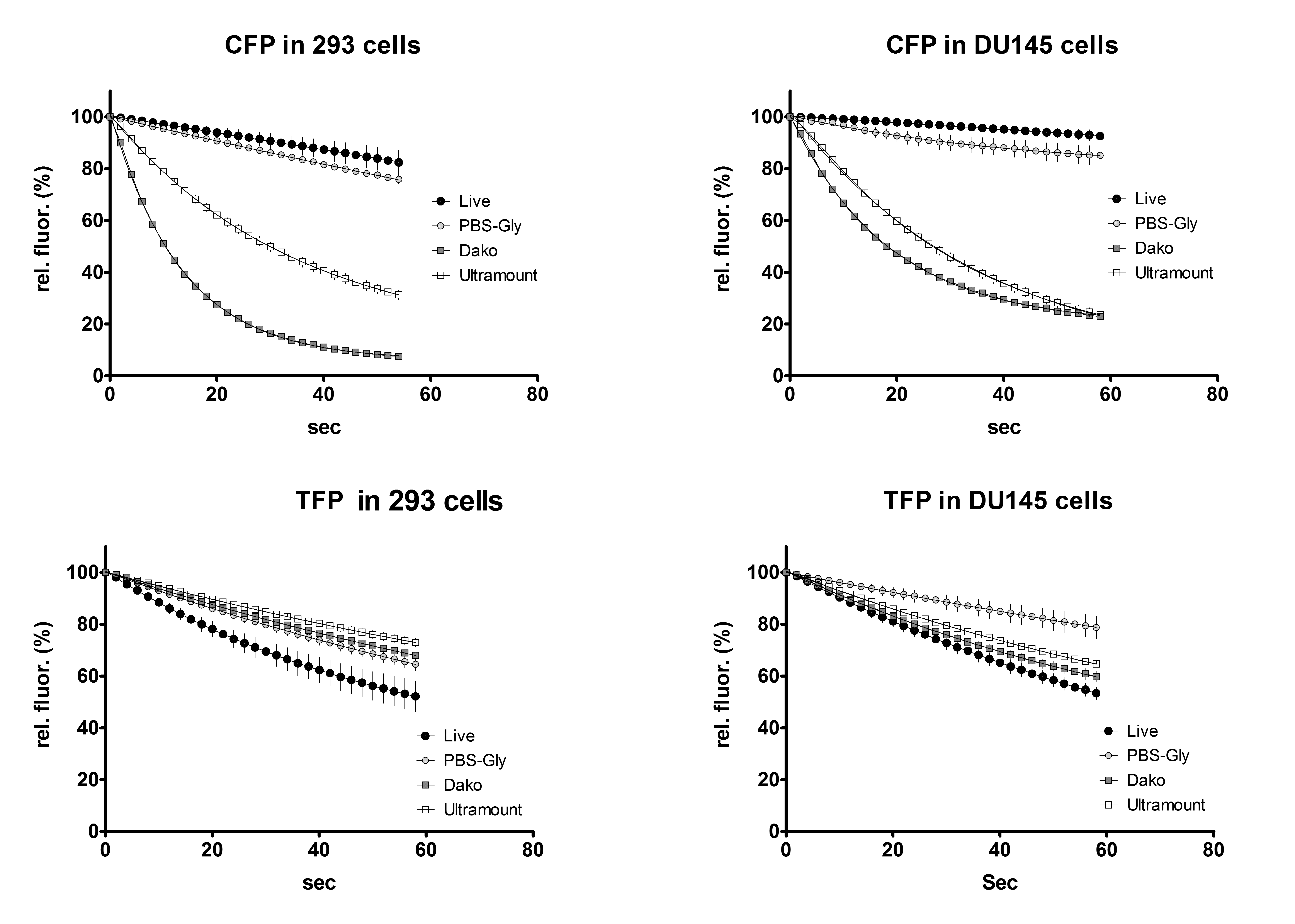

Supplement: Figure S2 — Bleaching characteristics is very similar in different cellular environments. 293 or DU145 cells were transfected with CFP (upper panel) or teal fluorescent protein (TFP, lower panel) as indicated and bleaching was recorded for live cells or for cells fixed with 4% paraformaldehyde in PBS and mounted in PBS/glycerol 1∶7 (PBS-Gly); in Dako fluorescent mounting fluid or in Thermofisher Ultramount. (TIF) [file pone.0018586.s002.tif]
